# The impact of artificial intelligence on the current and future practice of clinical cancer genomics

**DOI:** 10.1017/S0016672319000089

**Published:** 2019-10-31

**Authors:** Olivia Greatbatch, Alice Garrett, Katie Snape

**Affiliations:** 1 University College London, London, UK; 2 North East Thames Regional Genetics Service, Great Ormond Street Hospital for Children NHS Foundation Trust, London, UK; 3 South West Thames Regional Genetics Service, St George's University Hospitals NHS Foundation Trust, London, UK

## Abstract

Artificial intelligence (AI) is one of the most significant fields of development in the current digital age. Rapid advancements have raised speculation as to its potential benefits in a wide range of fields, with healthcare often at the forefront. However, amidst this optimism, apprehension and opposition continue to strongly persist. Oft-cited concerns include the threat of unemployment, harm to the doctor–patient relationship and questions of safety and accuracy. In this article, we review both the current and future medical applications of AI within the sub-speciality of cancer genomics.

## 1. Introduction

The concept of artificial intelligence (AI) is not a new one, but only in recent years has it garnered the levels of interest seen currently. In the form of consumer technology such as the virtual assistants built into most smartphones, AI has already deeply infiltrated our daily lives. The use of AI in our healthcare system is only in its primitive stages by comparison. The Topol Review recently explored how the National Health Service (NHS) can best exploit and adapt to the growing presence of technologies such as AI (Topol, 2019). Despite now being a regular subject of media attention, confusion and misunderstanding remain surrounding AI.

Therefore, for clarification, some key definitions are listed in Box 1.

Box 1. Key definitions of terms relating to artificial intelligence and cancer genomics.

**Intelligence**: ‘Goal-directed adaptive behaviour’ (Sternberg, 1982); that is, a measure of the ability to use one's cognitive functions to learn, adapt and maximize the probability of success of a given action, whether that is solving an equation or socially interacting.

**Artificial intelligence**: First defined as ‘the science of making intelligent machines’ (McCarthy, 1959). The aim of artificial intelligence is to emulate any aspect of intelligence in a way that either matches or exceeds what is attainable by humans alone.

**Robot**: ‘A machine which can carry out a complex series of actions automatically’ (Anon., 2010). Automation is not synonymous with artificial intelligence: artificial intelligence possesses the capacity to learn and develop without being explicitly programmed, whereas automation is dependent on pre-programmed input.

## 2. Applications of AI in cancer genomics

With an estimated 18.1 million new cancer cases recorded worldwide in 2018 (Ferlay *et al.*, 2018), the enormous burden placed upon healthcare infrastructure is unsurprising. Any means of alleviating the wide scope of medical, social and economic challenges would be welcomed.

In a field such as cancer genomics, there are several procedural levels at which AI could be incorporated to help do this:

1. Use of AI to present patient data in a way that increases the ease and efficiency of human interpretation.
2. Use of AI to recognize certain features and patterns, highlight these and suggest a course of action based upon existing manmade guidelines.
3. Use of AI-based systems that can learn from large volumes of data in order to become autonomous in their decision-making. In this instance, the input data and outcome are known but the process by which decisions are reached is opaque.

**Fig. 1.**
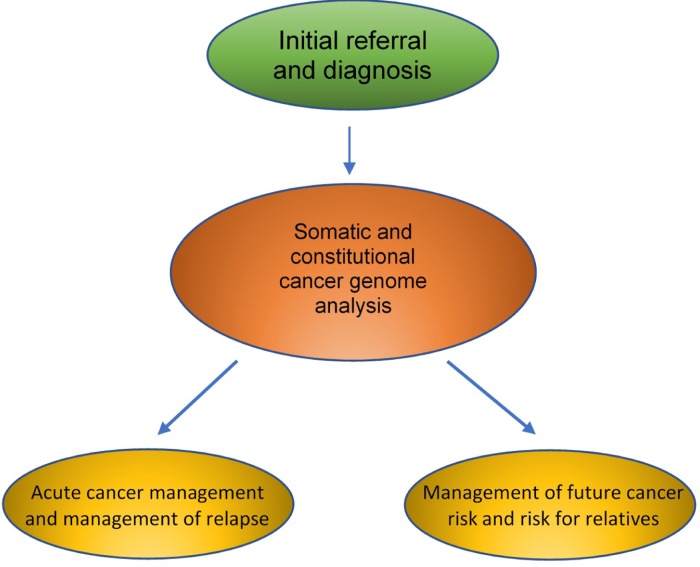
Areas of potential impact of artificial intelligence and genomics in the clinical care of a cancer patient.

With consideration to the recommendations proposed by the Topol Review, below we explore the potential uses of AI in cancer genomics, with reference to the stages in a patient's clinical pathway (Figure 1). This includes various real-world examples illustrating the various levels of complexity as listed above.

### 2.1. Initial referral and diagnosis of cancer

The usual first step along a cancer patient's journey is their contact with primary care. General practitioners (GPs) are responsible for the critical decision as to whether or not a case warrants referral for urgent investigation. Gathering all of the relevant information and making such important judgements within a 10-minute appointment is a challenging task for any clinician.

Any potential use of AI in this process is complicated by the diverse and complex nature of patient data, which are often contained in multiple individual electronic silos within the care record, or even, indeed, on a piece of paper. The Topol Review argues that fully digitizing and integrating these records is necessary for the implementation of AI, but how can this be achieved? The openEHR Foundation works to develop and implement the infrastructure of improved electronic medical records in order to help centralize patient data and improve continuity of care. However, the more we rely on technological solutions to NHS data issues, the more many of us become concerned regarding security. The WannaCry ransomware attack in 2017 cost the NHS £92 million and brought this concern to the fore (Harding, 2018). Clearly, developments in data protection must match the degree to which we become reliant on technology.

The Topol Review also proposes that AI will enable us to benefit from improved triage. For example, Babylon Health has already developed a chatbot tool that claims to do just this by asking questions about the patient's symptoms and then forwarding them to advice or a video call. However, many argue that its claims of accuracy and superiority over clinicians are unfounded. One publication concluded that there is limited evidence that Babylon is better than its human counterparts, and the Care Quality Commission's 2017 inspection reported that the company was failing to provide safe care (Fraser *et al.*, 2018).

Thus, to maximize the information-gathering and pattern-recognition benefits of AI, which could present the busy GP or oncologist with a clinically relevant overview of the patient in front of them to guide assessment, there are still huge challenges in data collection, collation and presentation that need to be overcome.

Following a referral, investigations are done to confirm or exclude a cancer diagnosis. These include biopsies, alongside histopathological, biochemical and radiological analyses. A proposed strength of AI would be the collation, assessment and pattern recognition of integrated datasets pertaining to these investigations to speed up and improve the accuracy of cancer diagnosis.

The Royal College of Pathologists is investing in the development of digital pathology (https://www.rcpath.org/discover-pathology/public-affairs/digital-pathology.html) and anticipates the future incorporation of AI into this. Likewise, radiology is also particularly ripe for integration of AI, such as through complex imaging pattern analysis (Hosny *et al.*, 2018). The Swiss start-up SOPHiA Genetics has developed a radiomics platform that aims to notice features in radiographic images that are missed by the human eye.

At present, cancer multidisciplinary team meetings contain a pathologist, radiologist and oncologist interpreting and discussing their various diagnostic tests. Proponents of AI suggest that the replacement of three experienced consultants with a single AI system may well be cost effective, as well as improving diagnostic accuracy, but proof of this in clinical practice remains elusive thus far.

### 2.2. Somatic and constitutional cancer genome analysis

Genomic analysis generates exceptionally large datasets. Ways of streamlining the analysis to allow easier recognition of clinically relevant variation are required, and such technology is already being used in real-world scenarios. Due to the fact that advances in genomic technologies generating wide-scale whole-genome data are a recent event, computational solutions for these analyses have always existed.

AI use in this type of analysis tends to use machine learning and pattern recognition to present input data in a format that lends itself to easier interpretation. The aforementioned SOPHiA Genetics developed a genetics AI that is the company's main platform and that uses knowledge-sharing between hospitals to help train its algorithms. This can locate and highlight mutations in cancer-linked genes within two hours of uploading raw genomic data, and 503 hospitals around the world are already using the technology, including several in the UK under the NHS (Macauley, 2018).

This optimization of data processing equally serves the interest of patients. The waiting period following genetic testing is associated with anxiety and other distressing psychological symptoms, which many patients find difficult to cope with (Godino *et al.*, 2015). Reducing the amount of time that this unpleasant process takes could help ease its mental toll.

### 2.3. Acute cancer management

#### 2.3.1. Personalized treatment plans

AI could also improve treatment decisions in the management of cancer patients. In one study, the time taken for the AI platform IBM Watson to analyse the genome of a brain cancer patient and suggest an appropriate treatment plan was compared with that taken by human experts. The experts took 160 hours, whereas Watson took a markedly shorter 10 minutes. Unfortunately, attempts to implement Watson in US hospitals fell massively short of expectations. There were reports of its recommendations providing no additional insight compared with those of the involved human doctors, and sometimes even including concerning inaccuracies (Loh, 2018). This highlights the importance of not rushing to introduce a technology without fully understanding its capabilities.

AI could also be integrated earlier on in the treatment process. For example, cancer researchers at the University of Montreal are currently using AI algorithms for the process of ‘deep learning’, which refers to a machine learning technique that teaches computers to learn by example, to investigate the genetic profile of cancer cells in acute myeloid leukaemia. This may have the potential one day to help determine the most appropriate course of treatment, although it is mostly still in the planning stage (Lemieux, 2018).

#### 2.3.2. Pursuing artificial empathy

The examples of AI mentioned so far supplement rather than replace doctors, with a human still having to input the data, often interpret the output and then relay it to the patient through conversation. Will the scope of AI grow beyond this in the future?

We all know that medical knowledge is not the only factor that makes a good doctor, whether real or digital. Empathy is so integral to medical practice that attempts are constantly made to analyse and quantify it. In one study, patients with the lowest levels of trust in their doctors had a treatment adherence rate of just 17.5% (Pearson & Raeke, 2000). In another, the patients of doctors who were ranked higher on the Jefferson Scale of Empathy experienced fewer acute complications that required hospitalization (Hojat *et al.*, 2013).

The Topol Review argues, however, that AI and robotics are best used to automate mundane tasks instead, so that the workforce can focus on the more ‘human’ elements of patient care. However, could it be feasible that AI will actually be used to emulate emotional intelligence and beyond?

One possible avenue of achieving this may be through cognitive developmental robotics, which involves the application of principles of human development to machines. One aspect of human empathy that has been applied to artificial intelligence is that relating to emotional contagion and motor mimicry. These terms encompass the phenomenon through which the emotions and behaviours of one individual directly trigger the same in others, such as wincing in response to observing someone else in pain (Asada, 2014). To achieve this, AI must replicate our ability to recognize patterns in people's behaviour when they are experiencing different emotions, even when we are unable to precisely describe the features seen (Mehta *et al.*, 2018). Pattern recognition from datasets of human expressions of emotions could perhaps be used to achieve this.

In humans and apes, empathy extends further beyond emotional empathy. We also possess cognitive empathy, which is the conscious drive to process and understand the emotional states of those around us. Cognitive empathy is yet to be emulated by AI and is the biggest barrier to true artificial empathy (Asada, 2014).

### 2.4. Management of future cancer risk

Screening, prevention and early detection for cancer are both clinically preferable and cost effective (Turnbull *et al.*, 2018). From a public health perspective, the media can have a large influence on public attitudes to hereditary cancer risk perception – so much so that the phenomenon has been termed the ‘Angelina Jolie Effect’, referring to the surge in enquiries and referrals following the actress's decision to undertake BRCA1 testing and a risk-reducing mastectomy (Evans *et al.*, 2014). However, when the phenomenon gives rise to inaccurate risk perception amongst the public, it can lead to patient anxiety and misuse of resources. This was a controversial area that most recently generated mainstream media debate following the personal reflections of the current UK Secretary of State for Health and Social Care, Matt Hancock, on his polygenic risk scores relating to his lifetime risk of prostate cancer.

#### 2.4.1. Risk prediction

AI can already help risk calculation. Data are put into online algorithms, which rapidly produce a figure indicating a particular risk, based on data modelling and inbuilt datasets of previous patients. The prevalence of these risk models is only likely to grow, with the incorporation of increasingly complex genomic data aiming to improve accuracy (Kim & Kim, 2018). However, useful as these tools might be, limitations remain.

Its distinctly non-human nature makes it easy to assume that AI is objective and unprejudiced. However, biases can be reinforced through machine learning if they are present within the datasets from which the AI learns (Char *et al.*, 2018). One study found that when their AI was fed large datasets of the English language written by humans, it learned word associations from these texts that mirrored societal associations. These ranged from more innocent patterns, such as flowers being connoted with pleasantness, to more sinister ones. With the researchers finding the AI linking European names with more positive associations versus certain African ones (Caliskan *et al.*, 2017), it is easy to visualize how a whole host of problems could arise for various population demographics in a healthcare setting. For example, if the inputs used to teach an algorithm are inherently biased towards the medical risks and needs of one group, then all other patients are at risk of an unequal standard of care.

Traditionally, information to input into risk models is usually obtained through taking a history from a patient. Patient understanding and comprehension can vary greatly, however. Clarification often needs to be sought by both parties, such as for apparent diagnoses of ‘stomach cancer’, which could mean anything from pancreatic cancer to lung cancer with liver metastases. Effective history taking is a complex process and does not follow a standard algorithm. Subtle signs may demonstrate that a patient might be withholding information, but an inability to pick up on this type of red flag is indeed one of the criticisms faced by the aforementioned Babylon chatbot.

#### 2.4.2. Risk communication and perception

Inappropriate interpretation of a calculated risk is another potential limitation of AI. The social, cultural and personal factors that may affect the significance of a risk for a particular patient cannot be easily incorporated into currently used algorithms. In addition, the way risk is presented to a patient has been shown to influence their risk perception (Keller & Siegrist, 2009). Wolfe *et al.* (2015) used a web-based intelligent tutoring system to educate patients about breast cancer genetics. Whilst they found this to be a cost-effective way of improving patient comprehension and decision-making compared with a non-interactive website, they concluded that this was no substitute for genetic counselling.

### 2.5. A threat to jobs?

The potential threat that AI poses against the utility of human employees is an argument at the forefront of its opposition, and the field of medicine is no exception to this. The Topol Review argues that there will be ‘significant changes to the roles and responsibilities of current NHS staff’ thanks to AI, but what form could these changes take?

Physical contact is needed to perform any sort of medical procedure, and even the most intelligent of digital software is, of course, very obviously not able to do this. However, robots have been propositioned as the hardware that could be controlled by AI to make this strange and intangible concept a reality. Most current healthcare robots, such as those in laparoscopic surgery, must be controlled by humans, and others have only a low level of AI influencing their physical actions. Nonetheless, the Topol Review anticipates that robots will be developed to exploit more advanced AI algorithms, enabling them to perform both manual and cognitive tasks.

However, the general public appears less receptive than Topol to the idea of a robotic coup of medical procedures, with only 23% of UK adults reporting that they are comfortable with the idea of intelligent machines performing medical operations (Timson, 2017). The importance of ensuring that the direction AI development takes aligns with the desires of the patients and public cannot be understated, meaning that AI-using robots should be developed with the end goal of supplementing, rather than replacing, the work of human professionals. Topol does also recommend that more employment opportunities should be generated to help develop these technologies, meaning that AI's growing role could actually generate more jobs in the NHS rather than threaten them.

### 2.6. Clinical governance

This review highlights the promising scope of innovation and enterprise in AI as a tool to improve the field of cancer genomics. Equally, however, issues and limitations that reach beyond the technology itself continue to persist. These issues must be addressed if AI is to be successfully integrated into the patient pathway.

The major increase in the use of genetic testing over the past decade has highlighted the inevitable fact that medical professionals are not infallible. In a review of one US legal database, more than 50 cases were identified in which healthcare providers were sued for failing to appropriately utilize genetic testing (Lindor & Marchant, 2011).

If a patient were to seek legal action following such an incident caused by an artificially intelligent machine, then who would be deemed responsible in this instance? Would it be the doctor who referred them, the software developer or the AI itself? It is often the case that the inputs and outputs of an algorithm can be seen, but the way in which the output was decided cannot. This is referred to as the ‘black box phenomenon’, and it further complicates these questions.

Indeed, a solution has not yet been developed, but there are multiple avenues that the issue of liability could take in such an instance. There is debate as to what extent an AI platform could be allocated the status of legal personhood, similar to how corporations can be allocated rights associated with human persons. Further, the people who manage the AI could be held responsible through vicarious liability, in the same way in which an employer is responsible for their employees’ actions.

Platforms under the spectrum of eHealth in the UK are included in the classification system of the Medicines and Healthcare Products Regulatory Agency. However, the growth of AI is already beginning to expose weaknesses in their framework. For example, the Babylon chatbot is counted as a Class I medical device, placing it in the same category as a wheelchair or stethoscope. This means that it does not need to pass a conformity assessment and is under minimal regulation.

## 3. Conclusion

The use of AI in healthcare continues to expand. Nonetheless, a dystopian future of clinicians ousted by a workforce of machines currently appears an unrealistic one. Ranging from data complexity to policy and patient trust, the areas of debate that must be tackled cannot be understated. Nonetheless, the rapid growth we are seeing in the development and use of AI is undeniable, and we must prepare ourselves for the inevitable changes we will see in our lifetimes. It is only by education, discussion and adaptation amongst both medical professionals and laypeople alike that we will best reap the benefits of AI in genomic medicine for everyone involved.
